# Loss-of-function of triacylglycerol lipases are associated with low flour rancidity in pearl millet [*Pennisetum glaucum* (L.) R. Br.]

**DOI:** 10.3389/fpls.2022.962667

**Published:** 2022-10-04

**Authors:** Rasika Rajendra Aher, Palakolanu Sudhakar Reddy, Rupam Kumar Bhunia, Kayla S. Flyckt, Aishwarya R. Shankhapal, Rabishankar Ojha, John D. Everard, Laura L. Wayne, Brian M. Ruddy, Benjamin Deonovic, Shashi K. Gupta, Kiran K. Sharma, Pooja Bhatnagar-Mathur

**Affiliations:** ^1^International Crops Research Institute for Semi-Arid Tropics (ICRISAT), Patancheru, Telangana, India; ^2^Department of Biotechnology, Osmania University, Hyderabad, Telangana, India; ^3^National Agri-Food Biotechnology Institute (NABI), Mohali, Punjab, India; ^4^Corteva™ Agriscience, Johnston, IA, United States

**Keywords:** pearl millet, *Pennisetum glaucum* (L.) R. Br., millet, milled flour, lipase, triacylglycerol, rancidity, shelf life

## Abstract

Pearl millet is an important cereal crop of semi-arid regions since it is highly nutritious and climate resilient. However, pearl millet is underutilized commercially due to the rapid onset of hydrolytic rancidity of seed lipids post-milling. We investigated the underlying biochemical and molecular mechanisms of rancidity development in the flour from contrasting inbred lines under accelerated aging conditions. The breakdown of storage lipids (triacylglycerols; TAG) was accompanied by free fatty acid accumulation over the time course for all lines. The high rancidity lines had the highest amount of FFA by day 21, suggesting that TAG lipases may be the cause of rancidity. Additionally, the high rancidity lines manifested substantial amounts of volatile aldehyde compounds, which are characteristic products of lipid oxidation. Lipases with expression in seed post-milling were sequenced from low and high rancidity lines. Polymorphisms were identified in two TAG lipase genes (*PgTAGLip1* and *PgTAGLip2*) from the low rancidity line. Expression in a yeast model system confirmed these mutants were non-functional. We provide a direct mechanism to alleviate rancidity in pearl millet flour by identifying mutations in key TAG lipase genes that are associated with low rancidity. These genetic variations can be exploited through molecular breeding or precision genome technologies to develop elite pearl millet cultivars with improved flour shelf life.

## Highlights

Non-functional triacyglycerol (TAG) lipases from low rancidity pearl millet lines had less degradation of TAG and lipid oxidation post-milling. Mutations in these genes can provide targets for developing pearl millet germplasm with extended flour shelf life.

## Introduction

Pearl millet [*Pennisetum glaucum* (L.) R. Br.] is the principal staple food for millions of people in arid and semi-arid regions of Asia and Africa, such as India and Nigeria. It is primarily grown in the driest regions due to its ability to tolerate and thrive under both continuous and erratic drought. This C4 plant requires low irrigation and resource management inputs when compared with other staples such as rice and wheat, effectively reduces atmospheric CO_2_, has high water use efficiency, and keeps drylands productive, ensuring food and nutritional security for smallholder farming communities (Nambiar et al., 2011). A powerhouse of nutrients like protein, minerals, vitamins, phytochemicals and antioxidants, pearl millet is gluten-free and contains high levels of polyphenols and other biologically advantageous compounds, thereby designating it as a “Smart food” (Tako et al., 2015). Its beneficial health properties such as lowering of fat absorption in the intestines and spike-free sugar release (low glycemic index) helps to reduce blood pressure, heart disease, and diabetes (Varsha and Narayanan, 2017).

Despite its nutritional profile and advantage over other cereals, pearl millet has remained unpopular due to the short storage life of the milled flour (Goswami et al., 2020). The rapid development of off flavors and taste within 5–7 days of milling poses a major hindrance for wider consumer acceptance, creates post-harvest waste, and causes drudgery to consumers, particularly women who are traditionally involved in food preparation. Over the last decade, pearl millet consumption has declined in urban areas and in commercial outlets (Rani et al., 2018), leading to lower incentives for its cultivation by smallholder farmers. To address rancidity, efforts have been made to develop post-harvest processing and pre-milling techniques to inactivate the biological components that lead to accelerated rancidity. However, these mechanical and physicochemical techniques have been shown to negatively influence the nutritional quality of both the micro- and macro-nutrients (Goyal et al., 2015). Therefore, to revive the importance of this nutri-cereal in dryland agriculture, further understanding of the biological processes that lead to the development of rancidity in pearl millet is urgently needed.

Pearl millet grain has a larger germ layer than other cereals, apart from maize (Taylor, 2004), and has a higher lipid content (5–7%), characterized by high levels of unsaturated fatty acids (Sharma et al., 2015). During milling of the whole grain, the bran and germ layers rupture and release endogenous lipases that commence hydrolysis of stored lipids and release of free fatty acids (FFAs). Lipases are more thermostable than lipoxygenases and show higher enzyme activity in low moisture conditions. Hence, inhibiting the initial formation of FFAs is critical for controlling seed lipid oxidation. While lipases are reported to play a primary role in the susceptibility of pearl millet to post-milling rancidity (Kumar et al., 2021), the underlying biochemical and molecular mechanisms have not yet been studied in detail, and to our knowledge the specific lipase(s) responsible have not been identified.

Here, we demonstrate that flour from pearl millet characterized as having low rancidity has lower expression of TAG lipases and is more resistant to TAG degradation and lipid oxidation than flour milled from high rancidity germplasm. Two TAG lipase genes (*PgTAGLip1* and *PgTAGLip2*) were identified in the low rancidity pearl millet line and contained polymorphisms that rendered the enzymes non-functional. These key TAG lipases may be used to develop seeds with greater post-milling shelf life, without affecting the beneficial nutritional profile of pearl millet.

## Materials and methods

### Plant material and growth

Initial screening experiments were performed on a representative panel of 12 inbreds and germplasm association lines (PMiGAP) developed by the millet breeding program at ICRISAT (Supplementary Table S1). These lines were grown at ICRISAT-Patancheru, India and harvested during the rainy season of 2018 and 2019, and analyzed in 2019 and 2021, respectively.

### Grain milling and storage conditions

Seed was stored in vacuum packed pouches in a cold room prior to analysis. 30 g of seeds from each sample were ground to fine powder in a custom-built burr-mill grinder or in a Cyclotec (Foss) grinding mill. The ground flours were spread into evenly distributed layers in lidless food grade trays (4 oz./118 ml) under accelerated storage conditions in incubator chambers at a temperature of 35 ± 0.1°C and 75 ± 3% relative humidity. Sub-samples of the flour were collected for the biochemical determination of acid value (AV), triacylglycerol (TAG) breakdown, FFA generation, and volatiles.

### Acid value

Total crude fat was extracted in a Soxhlet apparatus from flour using the standard method (AOAC, 1990) with a few modifications. Briefly, 5 g of flour was taken in a cellulose thimble which was suspended in a pre-weighed extraction beaker containing 100 ml petroleum ether (boiling point 60–80°C) and kept on a hot plate at 180°C until the sample started to boil. For the complete extraction of fat, the process was carried out for at least 90 min. Following removal of the solvent, the extracted oil was titrated against 0.1 N KOH using phenolphthalein as indicator and the end point recorded. The acid value was calculated using the formula:


$$\begin{array}{cc} \begin{array}{l} \mathrm{Acid value}\\ \left(\mathrm{mg}KOH/g\right) \end{array} & \begin{array}{l} =\left(\mathrm{Titrant volume}\times \mathrm{Normality of}KOH\times 56.1\right)\\ /\mathrm{Weight of sample} \end{array} \end{array}$$

where, 56.1 is the molecular weight of KOH (Hakoda et al., 2006).

### Quantification of lipids by GC-FID and HPLC-ELSD

Total lipid extractions were based on the method of Bligh and Dyer (1959). Briefly, lipids were extracted using 3 ml of 1:2 (v/v) chloroform/methanol by shaking at room temperature for 15 min. Phase separation was induced by the addition of 1 ml chloroform and 1.8 ml water. The lower lipid layer was collected, and samples were re-extracted twice with 2 ml chloroform. The lipid extracts were subsequently filtered and dried under a stream of nitrogen gas. In the 2021 experiment, the extraction method was modified to include an initial step to quench lipase activity by treatment with isopropanol heated to 75°C for 15 min; 0.01% BHT was added to all reagents. Neutral lipids were quantified by HPLC equipped with an ELSD (Evaporative Light Scattering Detector) using a cyanopropyl column (Luna 5 μm CN 100 Å 250 × 4.6 mm; Phenomenex) with hexane as mobile phase A and methyl tertiary-butyl ether (MTBE):isopropanol (95:5 v/v) plus 0.2% acetic acid as mobile phase B, with a gradient of 0 to 100% B, and re-equilibration of the column to 0% B. Standard curves of tri-C18:2 TAG and 18:2 FFA were run with each sample set to quantify total TAG and FFA as a percent of dry weight (% DW).

For quantitation of total fatty acids by GC-FID, 0.1 mg of 17:0 TAG was added to each sample as an internal standard. Acid catalysis was performed by adding 1 ml of 5% sulfuric acid in methanol followed by heating at 90°C for 1 h. Phase separation was induced by the addition of 1 ml of 1 M NaCl and 1 ml heptane. The upper heptane layer was transferred to a sample vial for analysis. FAME separation was performed on a GC system (Agilent 7890A) with an OmegaWax 320 column (Supelco) followed by FID analysis. The GC oven temperature was set at a starting temperature of 190°C, then increased to 240°C @ 5°C/min, with a total run time of 10 min.

### Analysis of volatiles by solid phase micro-extraction-GC-MS

Samples for each pearl millet line were subject to Solid Phase Micro-Extraction Gas Chromatography with Electron Impact Ionization Mass Spectrometry (SPME-GC-EI-MS) after 21 day of accelerated storage from the 2019 experiment. Triplicate flour samples (0.5000 ± 0.0001 g) of each line were weighed into 20 ml amber headspace vials that were brought to room temperature before incubation at 35°C for 1 h. Analysis was performed on an Agilent 7890 GC system equipped with an Agilent 5977B detector and a Gerstel Multi-Purpose Sampler (MPS Robotic; Gerstel) with automated SPME sampling capability. MS data files were pre-processed and analyzed with Genedata Expressionist software version 14.5^1^ (Asiago et al., 2012). Details on chromatography hardware parameters, software for processing and statistics is provided in Supplementary Methods (Appendix A).

### RNA isolation, cDNA synthesis and quantitative real time-PCR analysis

Pearl millet tissue samples (milled grain) were collected at 0 h, 6 h, 12 h, and 24 h of accelerated storage and used for total RNA extraction using RNeasy Plant Mini kit (Qiagen), and analyzed quantitatively and qualitatively using a NanoVue plus spectrophotometer (GE HealthCare). One and a half μg of RNA was used for cDNA synthesis using Superscript III (Invitrogen) and samples diluted 1:10 times were used as the template. Quantitative RT-PCR was carried out in 96-well optical reaction plates with a total reaction volume of 10 μl containing 0.5 μm of each primer (0.8 μl), cDNA (1.0 μl), Sensi Master Mix (2X) and dH_2_O added up to 3.2 μl. The quantitative real time-PCR (qRT-PCR) primers were designed using Primer3 software (v.0.4.0) with GC content of 40–60%, a T_m_ of 60–62°C, and primer length 20–25 nucleotides (Supplementary Table S3), for an expected product size of 90–180 bp. The qRT-PCR reactions were carried out using a standard thermal profile: 95°C for 10s, 40 cycles of 15 s at 95°C, 15 s at 61°C with fluorescent signal recording and 15 s at 72°C. After the 40th cycle, amplicon dissociation curves were measured by heating at 58 to 95°C with fluorescence measured within 20 min. All qRT-PCR data were obtained from three biological replicates with three technical replicates. Normalized expression was calculated with qBase+ software (Schmidt and Delaney, 2010) with reference genes *eukaryotic initiation factor4α* (*PgEIF4α*) and *malate dehydrogenase* (*PgMDH*; Reddy et al., 2015). List of all primers used for molecular studies is given in Supplementary Table S2.

Gene expression analysis was carried out using vegetative tissues (leaf, shoot and roots), seed developmental stages (embryo, milky seed, immature seed), and harvested grain flour under accelerated storage for 24 h. Lipid reserve mobilization studies were conducted on filter paper germinated seedlings sampled at regular intervals during 0–7 days after imbibition (DAI).

### Sequence analysis of the pearl millet TAG lipase genes

Rice lipase protein sequences were used as the query and searched in the latest version of the pearl millet genome (Varshney et al., 2017). Following removal of all duplicate and redundant sequences, the remaining sequences were analyzed for prediction of domains and motifs using the ExPASy PROSITE tool^2^ (Sigrist et al., 2012). Multiple sequence alignment was carried out with full-length protein sequences of PgTAGLips with ClustalW (MacVector (V17.1). Evolutionary relationship within lipases of pearl millet were investigated using the neighbor-joining algorithm of MacVector Software and the subcellular localization of proteins was predicted using the WoLF PSORT tool.^3^

### Molecular modeling and docking

Molecular models of PgTAGLips were generated using homology modeling server SWISS MODEL (Waterhouse et al., 2018) by utilizing the known structure of the template protein. Ramachandran plots were generated using PROCHECK (Laskowski et al., 1993) for validation of structures. Three dimensional structures of triglycerides and ester ligands substrate were retrieved using the PubChem database. Autodock 4.2 (Morris et al., 2009) was used for docking the triglycerides substrates to the lipase gene structures from the simulation. A grid box was prepared with dimensions 30 × 30 × 30 and centered on the ATP binding site with grid spacing of 0.478 Å. Ten docking runs were performed for each substrate with a population size of 50 and 15,00,000 energy evaluation. All other algorithm parameters used the default setting. The docked poses for each ligand were clustered with an RMSD tolerance of 1.5 Å. Predicting the possible protein-ligand interactions and the final pose of the substrate was selected based on its docking score.

### Gene cloning

Selected PgTAGLips were PCR amplified using cDNA as a template and cloned in pCR8/GW/TOPO TA Cloning vector (Invitrogen). All sequence confirmations were carried out with at least four to five colonies from each genotype. The vector backbone was trimmed, and sequences analyzed and aligned across genotypes using ClustalW to identify sequence variabilities.

### Yeast transformation

The cDNAs of PgTAGLip genes from selected genotypes were cloned in pYES2 vector under the control of galactose-inducible promoter and URA3 as a yeast selection marker. The yeast triple lipase mutant *∆tgl3∆tgl4∆tgl5* (ΔTGL; Klein et al., 2016) was used for TAG lipase heterologous characterization. All pYES2-PgTAGLip variants were transformed into ΔTGL according to the Ojha et al. (2021). Positive yeast transformants were selected on minimal synthetic defined (SD) base (Takara) media supplemented with complete supplement mixture without uracil (-Ura; Himedia, India) and further identified by colony PCR using conditions described above. Selected single yeast colonies were transferred into dropout base growth medium (Himedia, India) with 2% galactose and 1% raffinose, and incubated at 30°C for 24 h for lipid and FACS analysis.

### BODIPY^493/503^ staining and yeast lipid degradation analysis

Yeast ΔTGL cells transformed with different PgTAGLip variants were stained with BODIPY^493/503^ dye (4,4- Difluoro-1,3,5,7,8- Pentamethyl-4-Bora-3a,4a-Diaza-s-Indacene, Invitrogen) and used to quantify lipid degradation as an indication of lipase gene function. BODIPY dye was used to label the yeast TAGs according to Bhunia et al. (2021a). In brief, 2 μl of 1 mg/ml BODIPY stock solution (prepared in DMSO) was added to 1 ml yeast culture carrying different lipase variants and the samples were kept at 28°C for 20 min. Following the incubation period, yeast cells were centrifuged at 10,000 rpm for 10 min and the cell pellets washed thrice with PBS buffer and suspended in 1 ml of PBS buffer. Yeast cells were further diluted 1:10 in PBS buffer. Overnight cultures of yeast cells expressing TAG lipase were diluted into fresh medium containing cerulenin (10 μg/ml) and terbinafine (30 μg/ml) to block the *de novo* fatty acid biosynthesis and collected at 2 h, 4 h and 6 h intervals. Finally, BODIPY dye was used to label the yeast cells for flow cytometry analysis (Bhunia et al., 2021a). Labeled yeast cells collected at different intervals were diluted (1:10) in PBS buffer and used to measure the fluorescence intensity in a flow cytometer. The excitation and emission wavelengths were set at 493 nm and 503 nm, respectively.

### Flow cytometry analysis

Flow cytometry analysis was performed using high-speed flow cytometer, BD FACSAria Fusion (Becton Dickinson) to measure the uptake of yeast lipids according to Bhunia et al. (2021a). Every flow cytometry event was measured using side scatter (SSC) and LB fluorescence using FITC (530 ± 15 nm excited at 488 nm) filters. The mean fluorescence intensity values were analyzed using BD FACSDiva 8.0.1. The flow cytometer settings of all channels remained the same for all yeast cell sorting procedures.

### Statistical analysis

Determination of all chemical/biochemical parameters were carried out in triplicates and analyzed using Genstat. Several Log-linear hierarchical mixed models for TAG and FFA were fitted using the brms software package (Bürkner, 2017) in the R programming language. The model with the lowest leave-one-out information criteria (Vehtari et al., 2017) was chosen as the final model for inference. For more information see supplementary materials.

## Results

### Lipid degradation and off-flavor volatiles are increased in high rancidity pearl millet lines

**Figure 1:**
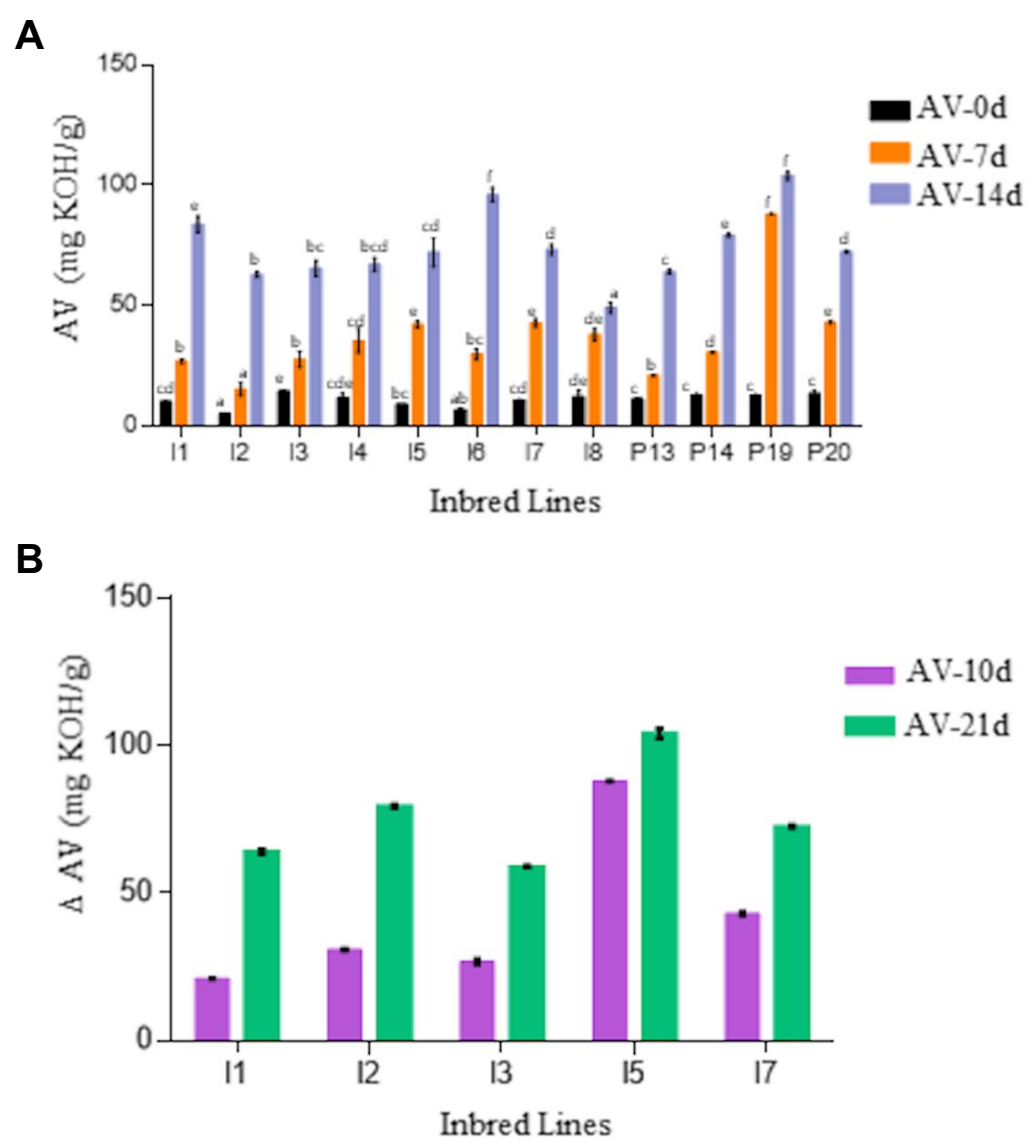
**(A)** Comparison of acid value (AV) of flour of 12 pearl millet inbred and germplasm association lines (PMiGAP) during 14-day accelerated storage conditions. The genotypes include ICMB-843 (I1); ICMB-88004 (I2); ICMB-95222 (I3); ICMB-81 (I4); ICMB-89111 (I5); ICMP-842 (I6); ICMB-863 (I7); ICMB-98222 (I8); IP5931 (P13); IP13840 (P14); IP 22419 (P19); IP6099 (P20). **(B)** Change in AV in the flour of selected contrasting lines on day 10 and 21. The data are represented as the mean ± standard error of three biological replicates. Variables showing *F*-value less than 0.05 were significant. Also, the means displaying non-matching lowercase letters were significantly different at *p* ≤ 0.05 by the Duncan test.

To examine the rancidification of pearl millet flour, acid values (AV) were determined in the oil extracted from flours of selected pearl millet lines subjected to accelerated storage conditions of high temperature and high humidity. Crude fat contents of the flours ranged between 4.2 and 7.2 wt% at the start of the experiment (day 0). The AV of freshly ground flours at day 0 varied between 5.15 to 13.6 mg KOH/g among the genotypes. Under accelerated storage flour conditions, AV increased significantly in all lines (Figure 1A), although the rates varied substantially between genotypes. For instance, while several lines (e.g., I6 and P19) had AV approaching 100 mg KOH/g after 14 day of accelerated aging, others (e.g., I2, I3, and I8) showed significantly lower AV (< 67.33 mg KOH/g). Based on these results, a subset of inbred lines with early onset of rancidity indicators were selected and re-evaluated in a 21 day experiment (Figure 1B). The I3 inbred line had lower AV, while the I5 and I7 lines had higher AV, indicative of greater FFA hydrolysis. A sensory panel also characterized the I3 line as less rancid (e.g., no odor, dry flour) and the I5 and I7 lines as more rancid (e.g., pungent, bad smell) after 21 day of accelerated aging (Supplementary Table S3).

**Figure 2:**
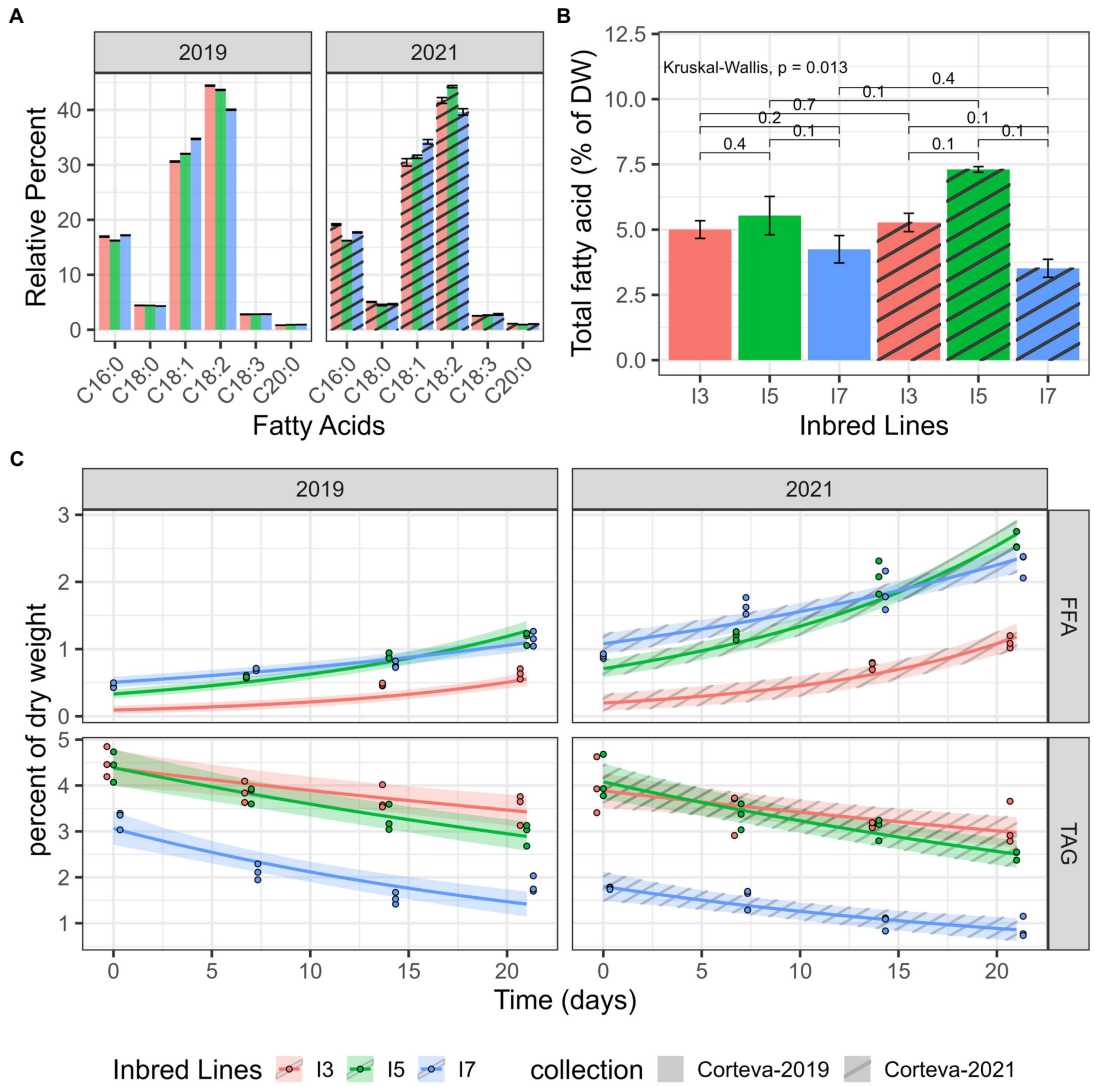
Lipid catabolism in milled flour under accelerated-aging conditions. **(A)** Fatty acid profiles obtained by GC-FID. **(B)** Total fatty acid quantitation by GC-FID, average over three replicates. Each brace corresponds to a Mann–Whitney test with the resulting value of *p* indicated above the brace. **(C)** TAG and FFA levels measured by HPLC-ELSD in two datasets (2019 and 2021). Log-linear hierarchical mixed models were utilized to assess the differences in TAG and FFA levels of these three inbred lines. Points represent observed data, lines represent posterior mean of the predictive distribution, and the ribbon corresponds to the 95% credible interval of the posterior mean predictive distribution.

The lipids from I3, I5, and I7 flours were analyzed to further characterize lipid hydrolysis. All three inbred lines had similar fatty acid profiles dominated by unsaturated linoleic and oleic acids (>75%), which are prone (particularly linoleic acid) to primary and secondary oxidation (Figure 2A). Additionally, pairwise comparisons using two sample Mann–Whitney tests across all fatty acids profiled showed no significant differences, suggesting that fatty acid composition is likely not the cause of differences in the propensity of the lines to develop rancidity. While there are some differences in total fatty acid values (Figure 2B), there were no significant differences between the lines (Kruskal-Wallis test and pairwise Mann–Whitney tests) in both experiment sets (2019 and 2021), and no significant differences in total fatty acid measurements were observed between the two experiments.

Two independent 21-day accelerated-aging experiments were performed to determine differences in lipid catabolism for the I3, I5 and I7 lines. The TAG and FFA levels were measured *via* HPLC from subsamples taken at each time point. For all lines, TAG levels showed a decreasing trend over time and a corresponding increasing trend in FFAs (Figure 2C). Pairwise posterior median differences on the log scale (averaged across the datasets) were computed to assess the significance of the differences between these three lines (Supplementary Table S4). The TAG values of the I7 line were significantly lower than the other two lines at all time points. The TAG values of the I3 and I5 line were not significantly different until day 14. All pairwise FFA levels were found to be significantly different except for the I5 and I7 lines at day 14. The I3 line maintained the lowest levels of FFA throughout the time course. Interestingly, the I7 line exhibited elevated FFA amounts at the onset of the time course across both datasets; it is unknown if I7 seed has elevated FFA prior to milling. Another 2021 dataset, using TLC/GC-MS, showed similar trends with increasing FFA especially in I7 and decreasing TAG levels over the time course (Supplementary Figure S1); however, this experiment was not quantitative and there was most likely sample loss. These results suggest that flour from the I7 line is more prone to TAG degradation during storage at high temperature and high humidity.

**Figure 3:**
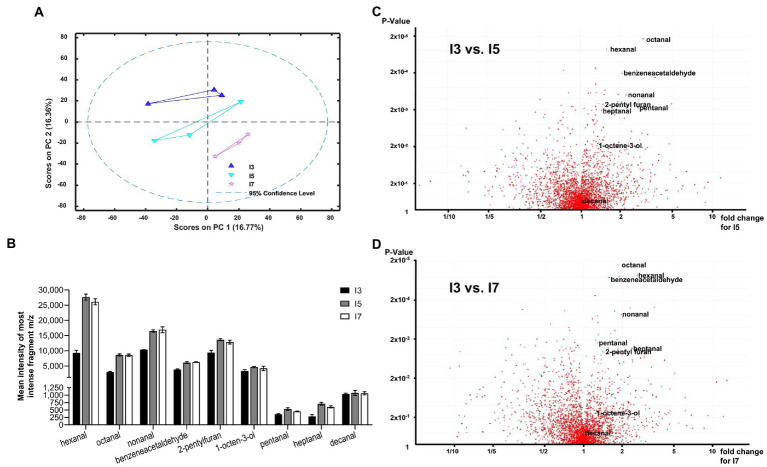
Volatile analysis of pearl millet I3, I5 and I7 lines using Solid Phase Micro-Extraction Electron Impact Ionization Gas Chromatography Mass Spectrometry (SPME-EI-GC-MS). **(A)** First and second principal components for processed auto-scaled datasets for the I3, I5 and I7 lines (blue triangles, aquamarine inverted triangles and pink stars, respectively). A 95% confidence range is indicated by a dashed line. **(B)** Most abundant fragment m/z total peak intensity bar charts for lowest value of *p* aldehydes (C_5_–C_10_) for the I3, I5 and I7 lines. **(C**,**D)** Volcano plots generated from 2 groups sample comparison tests for I3 vs. I5 **(C)** and I3 vs. I7 **(D)**. Most statistically significant aldehydes as measured by *value of p* are shown labeled. See Supplemental Table S5 for the detailed statistical analysis.

To confirm that the hydrolysis of TAG and the release of FFAs in flours is directly associated with the development of rancidity, the volatile chemicals released from the flours after accelerated storage were analyzed using SPME-GC-EI-MS. Principal component analysis of all auto-scaled components indicated that each line was distinct (Figure 3A); however, line I3 appeared to be markedly different from the lines I5 and I7 due to the presence of aliphatic aldehydes and other known markers of lipid oxidation (Figure 3; Supplementary Table S5). These data clearly indicate differential oxidation of fatty acids in the tested lines. From over 3,000 detected features in this analysis, four of the top 15 most statistically significant metabolites were aldehydes (Figure 3), which are known secondary oxidative products of unsaturated lipids. The I5 and I7 lines had significantly higher levels of hexanal, octanal, nonanal, and benzeneacetaldehyde compared to the line I3 in the headspace above flours exposed to 21 days of accelerated aging (Figures 3B,C). Since the fatty acid profiles for the three lines were similar (Figure 2A), the lines I5 and I7 seem to have greater susceptibility to rancidity than the I3 line due to higher amounts of FFAs and the oxidation of these FFAs.

### Polymorphisms in TAG lipases identified in the low rancid line are non-functional

**Figure 4:**
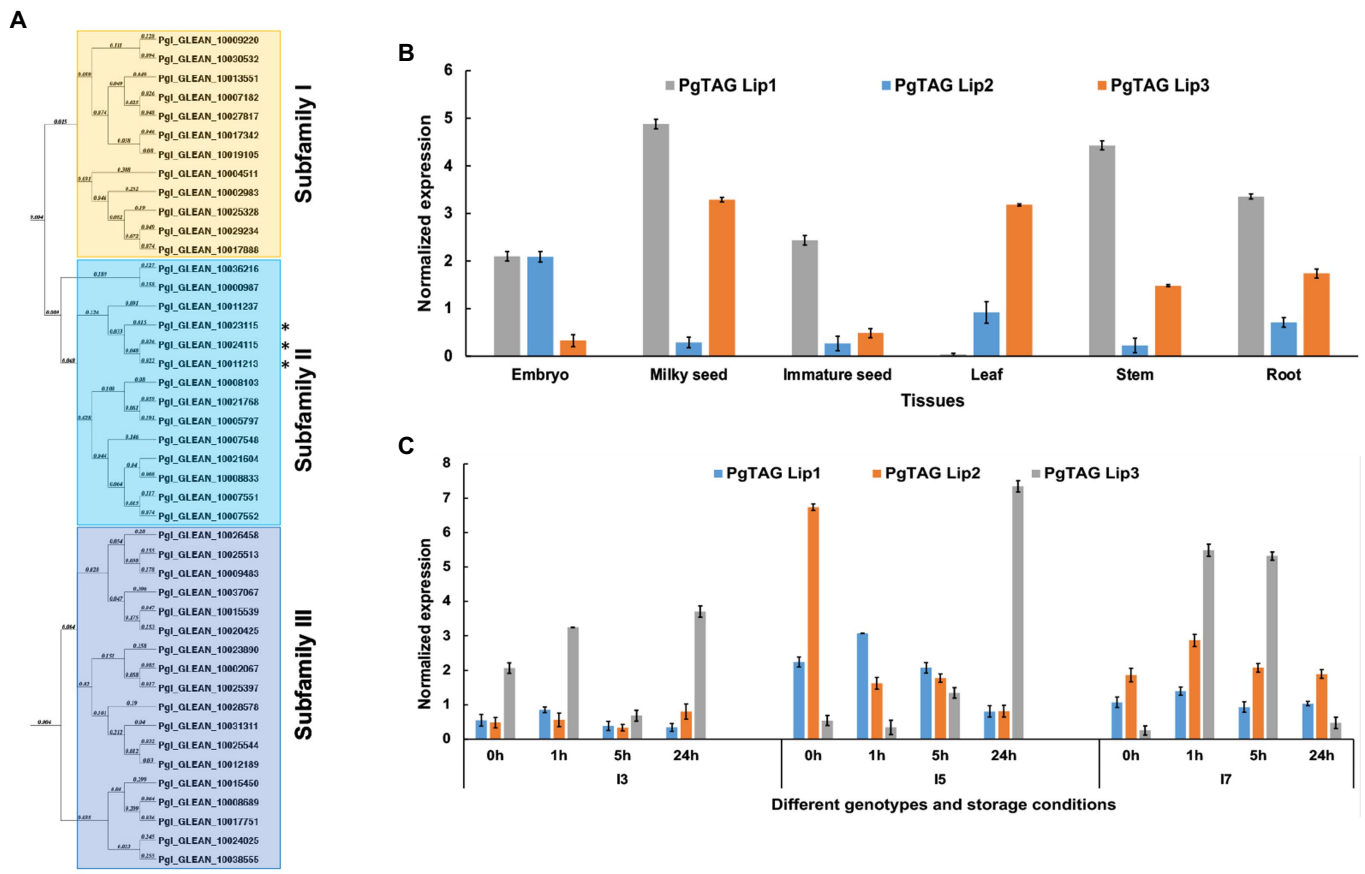
Phylogenetic relationships and expression analysis of the pearl millet lipases. **(A)** The phylogenetic tree constructed using MacVector software by the NJ method for PgTAGLips. Asterisks (^*^) denotes the genes used for further analysis: Pgl_GLEAN_10023115 was renamed Pg*TAGLip1*, Pgl_GLEAN_10024115 renamed Pg*TAGLip2*, and Pgl_GLEAN_10011213 renamed Pg*TAGLip3.* **(B)** Expression analysis of three selected PgTAGLips in the various developmental stages of pearl millet variety ICMB 9333. Data points represent the expression values, obtained after normalization against the reference genes *EUKARYOTIC INITIATION FACTOR4Α* (*PgEIF4α*) and *MALATE DEHYDROGENASE (PgMDH)*. Each data point represents the mean of three biological replications with standard error (±SE; representing the mean of three technical replicates). **(C)** Expression of the selected PgTAGLips in contrasting genotypes (I3, I5, and I7) under accelerated storage of milled flour. The x-axis represents the identities of flour samples stored at 0 h, 1 h, 5 h, 24 h. The y-axis indicates the normalized gene expression values against the reference genes Pg*EIF4α* and Pg*MDH*. Each data point represents the mean of three biological replications with standard error (±SE; representing the mean of three technical replicates).

The lipid and oxidative analyses suggest that these lines may have differences in TAG lipases. Our analysis identified a total of 44 lipase genes in pearl millet accessions in the International Pearl Millet Genome Sequence Consortium (IPMGSC) data. These lipases were divided into three major subfamilies based on sequence alignment and evolutionary relationships. Subfamily I and III characterized under PLD/PLC/esterase comprised 12 and 18 genes, respectively (Figure 4A). Subfamily II contained 14 genes, with a conserved LID domain and a catalytic triad, which are the characteristic features of TAG lipases. Subfamily II lipase gene and protein characteristics are summarized in Supplementary Table S6.

Based on the expression analyses of various plant tissues of the popular variety ICMB 9333 and 24 h accelerated storage of milled flour of the contrasting I3, I5, and I7 inbreds, three lipases were selected for further analyses. These lipases had higher similarity to the previously reported rice and Arabidopsis lipases (LOC_Os11g43510 and AT5G18640, respectively (Tiwari et al., 2016). The lipases Pgl_GLEAN_10023115, Pgl_GLEAN_10024115 and Pgl_GLEAN_10011213 were renamed PgTAGLip1, PgTAGLip2 and PgTAGLip3, respectively. Pg*TAGLip2* had high expression in immature embryos of ICMB 9333 compared to other tissues such as milky seed, immature seed, leaf, stem and roots (Figure 4B). While Pg*TAGLip1* expressed in most of the tissues, except leaf, with the highest expression at the milky seed stage, Pg*TAGLip3* showed higher expression in milky seeds and leaves (Figure 4B). During 24 h of accelerated storage of flours from the contrasting inbred lines, there were significant increases in the expression of all three genes in the flour from lines I5 and I7 compared to the flour from line I3 (Figure 4C). Interestingly, the expression levels of *PgTAGLip1* and *PgTAGLip2* were considerably lower in I3 flour compared to the flours of I5 and I7 (>1.5-fold) during the first 24 h (Figure 4C), suggesting that these lipase genes may be responsible for the differences in TAG degradation between these lines (Figure 2).

**Figure 5:**
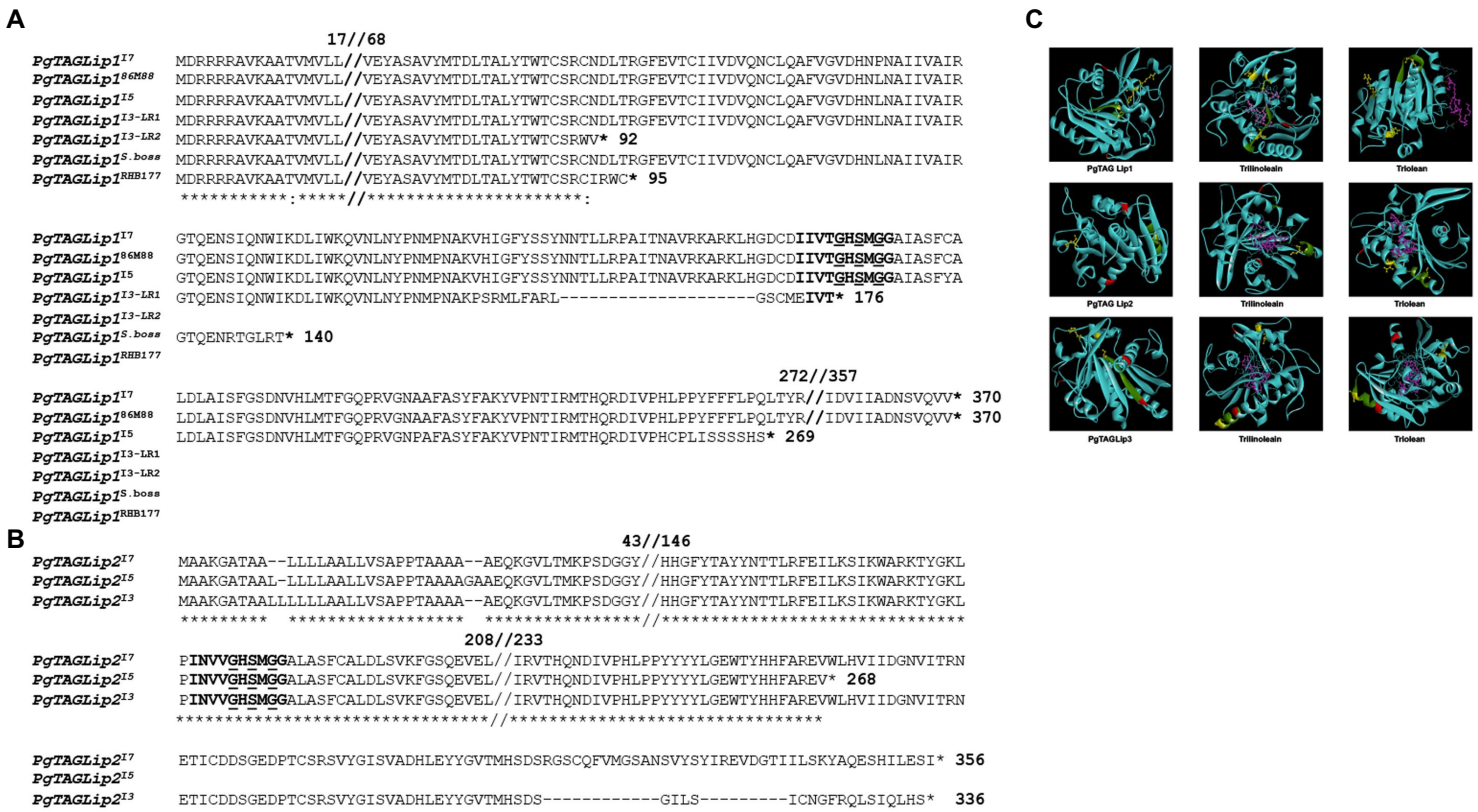
Structural variations of selected TAG lipases. **(A)** Comparative amino acid sequence alignment of Pgl_GLEAN_10023115 (PgTAGLip1) in high (I7, 86 M88, I5) and low rancidity genotypes (I3, Super Boss, RHB177). The LID domain is in bold, and the Lip serine active motif (GxSxG) is in bold and underlined. **(B)** Comparative amino acid sequence alignment of PgTAGLip2 in high rancid (I7 and I5) and low rancid (I3) lines. The LIP domain is bold and Lip serine active motif (GxSxG) is bold and underlined. **(C)** Predicted protein models and docking affinities of three PgTAGLips (PgTAGLip1, PglTAGLip2, and PgTAGLip3) with triolein and trilinolein. The predicted substrate binding is shown in a ball and stick model, with dashed pink lines denoting the hydrogen bonds. The putative LID domain GxSxG motif is in green, the catalytic triad is in yellow with ball and stick, the putative glycosylation sites are in red, and the ligand ball and stick is in pink.

Due to the differences in quantitative expression, the genetic sequences were analyzed for structural polymorphisms across genotypes. Sanger sequencing confirmed that the full-length coding regions of all three lipases carried a LID domain, an active site containing a serine GXSXG motif, and a catalytic triad (Supplementary Figure S2). However, the Pg*TAGLip1* gene sequences revealed polymorphisms among the lines I3, I5 and I7 (Figure 5A). To further confirm the correlation of these allelic variations with the observed and reported flour shelf life, additional genotypes (hybrids and inbred lines) based on a subset of the rancidity matrix were profiled for structural variations in Pg*TAGLip1*. The Pg*TAGLip1* gene in the I7 and high rancidity line 86 M88 formed a functional protein encoded by a 1,110 bp coding sequence, while the line I5 had a 807 bp coding region. Interestingly, the I3 line showed two transcript variants of Pg*TAGLip1* having fragment sizes of 528 bp and 276 bp (Supplementary Table S6); these polymorphisms were named LR1 and LR2, respectively. The LR1 transcript contained a stop codon within the LID domain that was expected to result in a loss-of-function of this gene, while the LR2 variant formed a much smaller peptide lacking the LID domain/active site and was also presumed to be non-functional (Figure 5A). In addition to the I3 line, other low rancidity lines Super Boss and RHB 177 also revealed mutations resulting in stop codons upstream of the LID domain at 420 bp and 285 bp, respectively (Figure 5A; Supplementary Table S6). It was intriguing to note that in contrast to the Pg*TAGLip2* transcript (1,068 bp) in line I7, single base variations and deletions up to 6 bp were observed in both lines I3 and I5 causing premature stop codons (Figure 5B; Supplementary Table S6). No sequence variations among the three tested genotypes were observed in Pg*TAGLip3*. These results provide further evidence that the maintenance of TAG, lower accumulation of FFA, and lower amounts of aldehyde volatiles in the line I3 are associated with allelic variations within the Pg*TAGLip1* and Pg*TAGLip2* genes.

To further support our annotations, molecular docking was used to predict the affinity/specificity of these pearl millet TAG lipases to several triacylglyceride ligands (Supplementary Table S7). *Rhizomucor miehei* lipase (RML; PDB id: 3TGL) was used as a template to generate the three-dimensional models and shared a 32–55% peptide identity with the PgTAGLips. The modeled structure when superimposed with the template (PDB id: 3TGL) showed an overall root mean square deviation (RMSD) of 1.66 suggesting close structural similarities among the modeled and template structures. Geometrical aspects of the modeled structures revealed that >70% of the modeled secondary structure was favorable and based on the predicted Ramachandran plots, only 0.9% of residues fell in the disallowed region, thereby confirming the high quality of the modeled structures. Protein-ligand docking revealed that, PgTAGLip1, PgTAGLip2, and PgTAGLip3 had very high affinities/specificities for triglycerides containing unsaturated fatty acid residues, suggesting that these lipases have a predisposition for degrading TAG containing linoleic and oleic fatty acids in pearl millet flour (Figure 5C; Supplementary Table S7).

**Figure 6:**
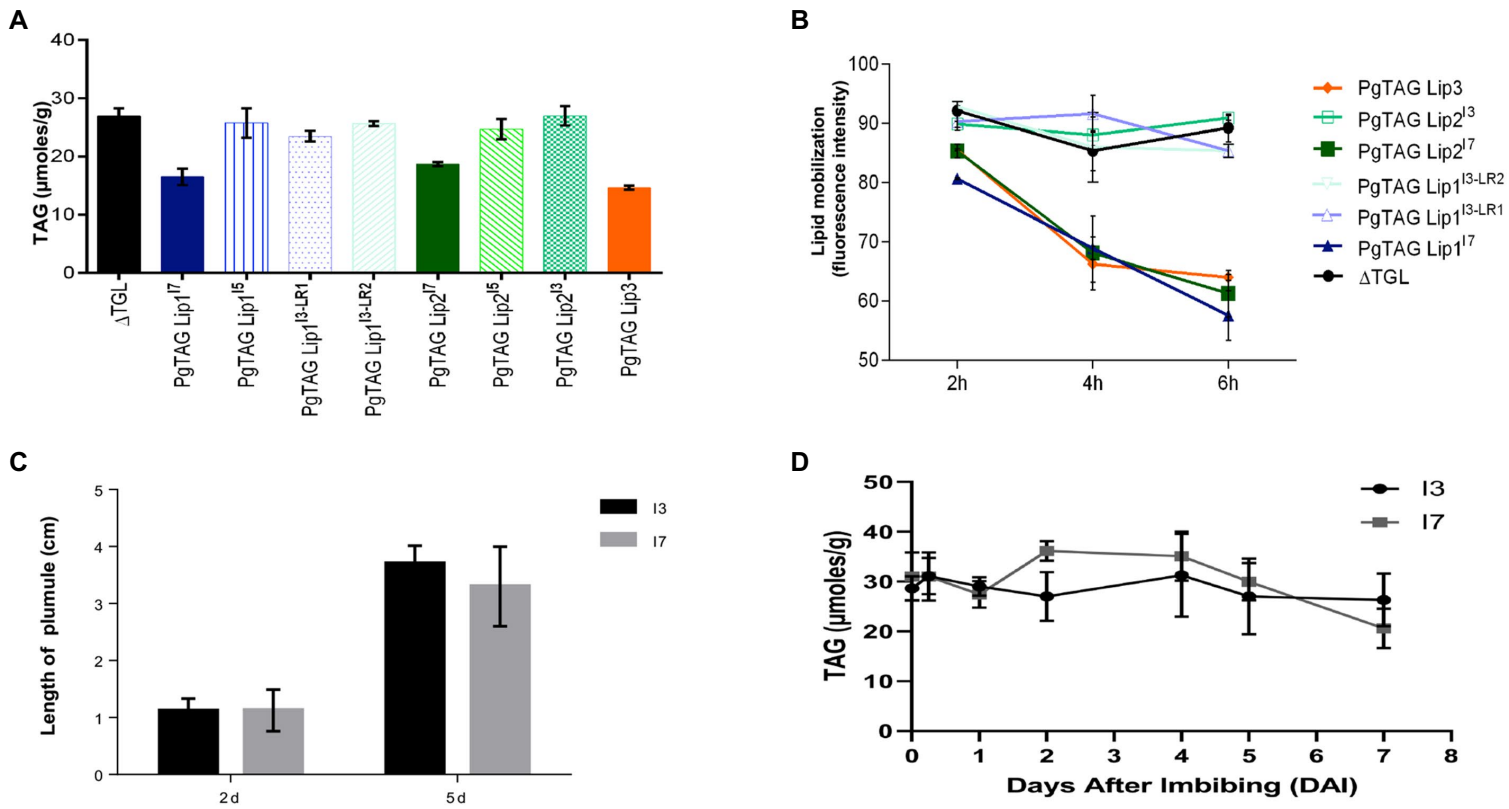
Functional validation in yeast expression system and seedling growth of the contrasting pearl millet seeds lines during germination **(A)** GC-MS TAG analysis of yeast cells expressing TAG lipase variants. Each data point represents the mean of three biological replications with standard error (±SE; **B**) *in vivo* mobilization of TAG reserve in yeast triple lipase mutant expressing TAG lipase variants from 2 h to 6 h. Each data point represents the mean of two biological replications with standard error (±SE). **(C)** Seedling establishment of I3 and I7 lines. **(D)** TAG mobilization during germination and post-germinative growth. Each data point represents the mean of three biological replications with standard error (±SE).

To determine if the PgTAGLip allelic variants were functional, they were assessed for complementation in a lipase-deficient yeast mutant. The yeast triple lipase mutant *Δtgl3Δtgl4Δtgl5* (ΔTGL) lacks all three endogenous lipases. Pg*TAGLip1*, Pg*TAGLip2*, and Pg*TAGLip3* genes were cloned from the lines I3, I5 and I7 and transformed into ΔTGL. To indicate the germplasm source and genotypic identity of allelic variants (mutants), superscripts were added to the gene names, e.g., PgTAGLip1^I7^, PgTAGLip1^I3-LR1^, etc. The total amount of TAG from the transformed yeast cells was analyzed by GC-MS. Yeast cells expressing full-length (non-truncated) PgTAGLip1, PgTAGLip2 and PgTAGLip3 accumulated less TAG (16.51, 18.72 and 14.65 μmol/g, respectively), when compared to the ΔTGL control (26.93 μmol/g; Figure 6A). The truncated allelic variants PgTAGLip1^I3-LR1^, PgTAGLip1^I3-LR2^, PgTAGLip2^I3^ and PgTAGLip2^I5^ accumulated comparable TAG content to ΔTGL (Figure 6A) confirming that these truncations are non-functional.

To corroborate the lower amounts of TAG from the truncated PgTAGLip variants, mean fluorescence intensity of yeast TAGs stained with BODIPY^493/503^ were analyzed by flow cytometry. There was a significant decrease in fluorescence intensity in the populations of yeast cells (P3) expressing full-length lipase genes compared to the control (ΔTGL expressing empty vector). The yeast cells carrying non-truncated versions of PgTAGLip1, PgTAGLip3, and PgTAGLip2 cloned from the I7 line had lower intensities (64.25, 58.10, 54.15% respectively), reflecting TAG breakdown. In contrast, the cells expressing truncated variants PgTAGLip1^I3-LR1^, PgTAGLip1^I3-LR2^, PgTAGLip2^I3^_,_ and PgTAGLip2^I5^ showed higher fluorescence intensities, *viz.* 82.65, 84.75, 82.55, and 89.8%, respectively (Supplementary Figure S3A). Hence, these results demonstrate that all the truncated allelic variants (PgTAG Lip1^I3-LR1^, PgTAG Lip1^I3-LR2^, PgTAG Lip2^I3^ and PgTAG Lip2^I5^) had reduced functionality.

### TAG lipases with loss-of-function mutations do not mobilize TAG and have no impact on seedling establishment

The reduction in TAG content in yeast strongly suggested that all three non-truncated PgTAGLip exhibit lipolytic function. To demonstrate that deleterious mutations in the variants affect their function, time-dependent turnover of TAG was estimated during the yeast growth (up to 6 h) and measured using flow cytometry. Results indicated that the ΔTGL mutant and truncated variants PgTAGLip1^I3-LR1^, PgTAGLip1^I3-LR2^, PgTAGLip2^I3^ and PgTAGLip2^I5^ did not mobilize TAG products. In contrast, the non-truncated versions (cloned from the line I7) were significantly active in mobilizing TAG products during the 2 h to 6 h time course (Figure 6B).

To determine if the observed differences in lipid degradation between the lines I3 and I7 had any effect on seedling establishment, plumule elongation and lipid mobilization were measured during germination. No differences were observed in post-germination seedling establishment or growth (Figure 6C), indicating that while the mutations in both candidate lipases may be useful for minimizing TAG hydrolysis, these enzymes are not essential for germination. Additionally, there were minimal differences in TAG mobilization between the lines I3 and I7 during germination (Figure 6D); most likely other TAG lipases are involved in mobilizing TAG for germination. Taken together, these results suggest that mutations in PgTAGLip1 and PgTAGLip2 contribute to lower amounts of FFA accumulating in seeds post-milling. Therefore, allelic variations in these genes may provide an opportunity to decrease the amount of FFA (and thus their oxidation), thereby alleviating the propensity of rancidity development in pearl millet flour.

## Discussion

Considered as the “mother of all problems” in pearl millet, flour rancidity is the most important intervention required for ensuring a sustainable demand for this nutri-cereal. While stabilization techniques have been effective in minimizing rancidity to some extent, only large-scale processing would justify their economic feasibility, which may not be practical in rural agricultural communities. The improved stability of shelf life following stringent treatments suggests that biological enzymatic-driven processes drive the generation of off-flavor volatiles in pearl millet flours. Thus, genetic improvements are a promising alternative to physical stabilization techniques for extending the storage life of flour.

### Biochemical analyses suggest rancidity of pearl millet flour is due to lipid catabolism

A set of 12 pearl millet genotypes based on an earlier study (Majumdar et al., 2016) were investigated for identifying genotypes with contrasting susceptibility to rancidity. The change in AV, an indicator of esterified lipid hydrolysis and the presence of FFA over 10 days from seed grinding, was used to categorize the pearl millet lines. Contrasting inbred lines were categorized as low AV (I3), high AV (I5), and intermediate AV (I7) that were further confirmed by sensory panel analysis. Clear differences between the selected lines were observed in the amounts of TAG and FFA present in the seeds at the time of grinding through the accelerated-aging treatment, which were in broad agreement with the initial AV studies. Further analysis after 21 days of accelerated storage showed that aldehydes and other markers of fatty acids, primary and secondary oxidation (1-octen-3-ol and 2-pentyl furan) were the dominant discriminators in comparisons between the headspace volatiles of the flours from lines I3 vs. I5 and I3 vs. I7. Not only are these volatile chemicals the key markers of oil oxidation (Franklin and Mitchell, 2019), but they are also associated with the development of rancidity in foods rich in polyunsaturated fats, including seeds and flours (Gorji et al., 2019). Other, non-lipid related off-flavor metabolites such as 2-acetyl-1-pyrroline and apigenin have been identified in high moisture (30%) pearl millet flours within 15 h of grinding and wetting (Reddy et al., 1986; Seitz et al., 1993) where both have been associated with a mousy acidic odor detected in millet flours. Mass spectral searches of the volatile chemistry profiles of the 21-day aged flours analyzed in this study using the NIST-AMDIS application failed to detect these two compounds, thereby indicating that they were not principal factors in this material and that products of lipid oxidation were closely associated with flour rancidity.

These results indicate that differential hydrolysis of esterified lipids is linked to the release of FFA and is closely associated with the development of rancidity under accelerated-aging conditions. Although PgTAGLip1 and PgTAGLip2 in the I5 line are prematurely truncated, the flour from the I5 line accumulated more FFAs. This discrepancy could possibly be due to functional redundancy among TAG lipases. We presume that the amphipathic polyunsaturated FFAs are more prone to oxidative degradation once released from lipid bodies and membranes resulting in the generation of short chain aldehydes and other secondary oxidation products characteristic of oil rancidity (Bhunia et al., 2021b). Considering that the fatty acid profiles for the three lines were similar, these results indicate that the lines I5 and I7 had higher propensity for rancidity than the line I3 due to higher amounts of FFAs and the subsequent oxidation products. Furthermore, this direct association between TAG degradation and off-flavor volatiles suggest that there may be transcript or genomic differences in the TAG lipases between these pearl millet lines.

### Non-functional mutations in PgTAGLip1 and PgTAGLip2 are associated with low rancidity

Three PgTAG lipases with close homology to those reported from *O. sativa* and *A. thaliana* (Tiwari et al., 2016) were found to have differences in transcript levels and sequence between the three tested pearl millet lines. Expression analyses showed that Pg*TAGLip1*, Pg*TAGLip*2 and Pg*TAGLip3* were expressed in stored flour, with line I3 having the lowest expression of Pg*TAGLip1* and Pg*TAGLip2*, suggesting a catalytic role in the degradation of triacylglycerols (TAGs) and FFA generation even after milling. Further insights connecting these TAG lipases to the differential susceptibility to rancidity in the I3, I5, and I7 lines were revealed through sequence analysis where mutations were observed in the coding sequences of Pg*TAGLip1* and Pg*TAGLip2* of lines I3 and I5. Structural polymorphisms (SNPs and deletions) in Pg*TAGLip1* and Pg*TAGLip2* led to premature stop codons that terminated the Pg*TAGLip* gene products before or close to the catalytic center.

TAG lipases contain a catalytic serine-aspartate-histidine triad that is required for activity (Wada and Murata, 2009; Tiwari et al., 2016). A LID domain covering this active site is present in lipases and is critical for the interaction with TAG substrates (El-Kouhen et al., 2005). Our study revealed that in the I7 high rancidity line, PgTAGLip1 had an intact LID domain and catalytic triad, while in the I5 line, PgTAGLip1 lacked the histidine residue (Supplementary Table S8). In contrast, the low rancidity I3 line altogether lacked these active site motifs in this gene. Interestingly, while premature truncations were apparent in the *PgTAGLip2* gene in lines I3 and I5, the catalytic center was intact in all three lines. While our study did not confirm the effect of truncations on the enzymatic activity, the protein docking of various ligands (triglycerides) with both PgTAGLip1 and PgTAGLip2 predicted very high affinity/specificity for triolein and trilinolein substrates. These substrates are most likely some of the endogenous substrates, since oleic and linoleic fatty acids are major components of pearl millet flour (Zaplin et al., 2013). In addition to the Pg*TAGLip1* polymorphisms uncovered in line I3, detection of similar mutations in other low rancidity lines (Supplementary Table S8) like Super Boss and RHB177, further imply that mutations in this TAG lipase are the primary cause of rancidity in pearl millet. These findings are in line with previous reports where mutations in the serine residue in the GXSXG motif of *Pseudomonas aeruginosa* patatin-like phospholipase and yeast TAG Lipase 4 with an alanine residue almost demolishes lipase activity (Kurat et al., 2006). Similarly, complete deletion of the GXSXG motif in mouse phospholipase A2 (PLA2) almost eliminates its enzymatic activity (Wada and Murata, 2009). A yeast lipase-deficient system can be used to test the functionality of heterologous lipase genes (Bansal et al., 2021). Since the yeast storage lipids are structurally and functionally similar to seed storage lipids, it serves as an ideal *in vivo* system to ascertain the role of candidate lipases in TAG hydrolysis (Jacquier et al., 2013). A comparison of the truncated lipases from the line I3 (Pg*TAGLip1*^I3^ and Pg*TAGLip2*^I3^) with the non-truncated lipases from the line I7 (Pg*TAGLip1*^I7^ and Pg*TAGLip2*^I7^) revealed that the lipase variants in line I3 were less functional in mobilizing yeast storage lipids. These results demonstrate that rendering PgTAGLip1 and/or PgTAGLip2 non-functional through mutations in the active site motifs can provide a mechanism for maintaining TAG levels and decrease off-flavor volatiles in the flour.

### TAG lipase polymorphisms can be used to develop pearl millet varieties with low susceptibility to rancidity

For the application of mutating TAG lipases to alleviate the rancidity of pearl millet flour and increase shelf life, physiological and agronomic effects must be minimal. Lipases are important in the catabolism of lipid reserves, especially during germination and post-germination seedling establishment (Eastmond, 2006). Our results show minimal changes to seedling establishment and TAG mobilization in the I3 line containing polymorphisms in Pg*TAGLip1* and Pg*TAGLip2*, thereby suggesting that these genes are not essential for germination or post-germination growth. While both of these TAG lipases may be useful for TAG hydrolysis, since the variants do not mobilize TAG, there are most likely functional redundancies among PgTAGLip enzymes.

## Conclusion and future prospects

Specific polymorphisms in pearl millet lipases have been identified that can be used for breeding high yielding hybrid pearl millet varieties with prolonged flour shelf life. Our study also demonstrates strategies for future chemical mutagenesis or CRISPR-based approaches to create loss-of-function mutations in Pg*TAGLip1* or Pg*TAGLip2*, thereby mitigating rancidity in elite milled pearl millet germplasm. Increasing the storage life of flour from this nutritious grain offers tremendous opportunities for primary and secondary processing, creating markets and enhanced profits for smallholder farmers in South Asia and Sub-Saharan Africa.

## Data availability statement

The datasets presented in this study can be found in online repositories. The names of the repository/repositories and accession number(s) can be found in the article/Supplementary material.

## Author contributions

RA: investigation and validation. PR: methodology, original draft, and analysis. RB: methodology, investigation, and review and editing. KF: investigation and review and editing. AS: investigation and validation. RO: investigation. JE: conceptualization, methodology, investigation, and review and editing. LW: investigation, analysis, and review and editing. BR: data curation and visualization. BD: data curation. SG: seeds and review. KS: conceptualization, analysis, original draft, review and editing, and project administration. PB-M: conceptualization, funding acquisition, methodology, formal analysis, original draft, supervision, and project administration. All authors contributed to the article and approved the submitted version.

## Funding

RA and RB acknowledge fellowship support from the Department of Science and Technology in the form of INSPIRE fellowship (DST/INSPIRE/03/2018/000417) and INSPIRE Faculty award (DST/INSPIRE/04/2017/000484), respectively. PB-M acknowledges the financial support from the CGIAR Research Program on Grain Legumes and Dryland Cereals (CRP-GLDC) supported by CGIAR Fund Donors.

## Conflict of interest

KF, JE, LW, BR, and BD were employed by the company Corteva™ Agriscience.

The remaining authors declare that the research was conducted in the absence of any commercial or financial relationships that could be construed as a potential conflict of interest.

The reviewer AR declared a past co-authorship with the author SG to the handling editor.

## Publisher’s note

All claims expressed in this article are solely those of the authors and do not necessarily represent those of their affiliated organizations, or those of the publisher, the editors and the reviewers. Any product that may be evaluated in this article, or claim that may be made by its manufacturer, is not guaranteed or endorsed by the publisher.

## Footnotes

^1^ www.genedata.com

^2^ http://prosite.expasy.org/

^3^ http://psort.hgc.jp/form.html

## Supplementary material

The Supplementary material for this article can be found online at:

https://www.frontiersin.org/articles/10.3389/fpls.2022.962667/full#supplementary-material
