# Supplementary material for: Loss-of-function of triacylglycerol lipases are associated with low flour rancidity in pearl millet [Pennisetum glaucum (L.) R. Br.]
Source: Front Plant Sci. 2022 Oct 4;13:962667. doi: 10.3389/fpls.2022.962667 (PMC9577237; doi:10.3389/fpls.2022.962667)
Supplement: Supplementary file 1 [file Data_Sheet_1.pdf]

Supplementary Material

Supplementary Methods

Sensory evaluation of flours under accelerated storage

A preliminary sensory panel of basic attributes to profile the aroma, flavor, and taste of flours from the selected inbred lines was carried out at ICRISAT (Table S3). The samples were presented to a panel of six assessors randomly in replicates of 5 at regular intervals during accelerated storage (up to 21 days), to characterize the differences in aroma and taste. The assessors were advised to not differentiate by color. The responses were summarized based on the frequency of descriptors. Ground coffee powder was used to clear the olfactory sensors between sample evaluations.

Lipid quantitation by TLC/GC-MS

Total lipids from pearl millet flour and yeast cells were extracted according to Sinha et al. (2020) and were separated through thin layer chromatography (TLC). For GC-MS analysis, FAMEs of TAG and FFAs were prepared from the individual lipids using 4% (v/v) sulfuric acid in methanol along with 0.2 mL toluene and 10 µg of heptadecanoic acid (17:0, Sigma, USA) as an internal standard. FAMEs were quantified using Agilent Technologies Model 7890B gas chromatograph equipped with 7000 GC/MS triple quad detector and a non-polar DB-5 capillary column (J&W 123–5533, 30 m x 320 µm × 1 µm) according to Sinha et al. (2020). The FA concentrations were determined using the known concentration of 17:0, represented as a percent of dry weight (%DW) and µmoles/g dry weight (µmol/g DW).

Statistical Modeling

Separate models were fit for TAG and FFA. Several models were fit all of which included line as a fixed effect to allow for inference. Other factors considered for inclusion in the model are time (measured in days since start of experiment; 0, 7, 14, and 21 days) and an indicator from which experiment the data are from (i.e., collection: Corteva-2019, Corteva-2021, and NABI-2021). A random effect was included to account for the correlation among technical replicates. Models

including all combinations of the fixed effects and interactions of the fixed effects were fit. The FFA values in two of the collections (Corteva-2019 and Corteva-2021) exhibited left censoring of several values due to lower levels of detection. These values were included in the analysis as censored values with the censoring threshold set to the minimum of the non-censored observations from these two collections.

The models were fit using the brms software package fit using the brms software package (Bürkner, 2017) in the R programming language (R Core Team, 2021). Models were compared to select a model which balances parsimony and fit to the data by comparing and selecting the model with the lowest leave-one-out information criteria (Vehtari et al., 2017). The models selected for both TAG and FFA analysis included the time component (allowing for changing TAG and FFA levels over time) as well as an interaction between time and line type (allowing varying trends between each line). The model selected for TAG additionally included collection as a covariate (allowing for varying initial TAG levels), while the FFA model included collection as a covariate as well as the interactions between collection with time and line (allowing for varying trends between the lines across the three collections).

Solid Phase Micro-Extraction-GC-MS parameters & data analysis

For all samples and controls, the headspace vials were agitated (250 rpm; 70˚C) for 5 min prior to SPME (Supelco 57298-U; Gray) headspace sampling for 20 min. With the inlet (0.75 mm ID liner; Restek) set at 250 °C in a splitless mode, the fibre was exposed to start the cycle. After 5 min in the inlet the split vent was opened (30mL/min) and was retained until the next cycle began. Chromatographic separation was achieved on an Agilent Ultra-1 column (50m x 0.32, 0.52u film thickness; helium carrier gas; 1.1 mL / min constant). The column oven was maintained at 40 °C for the first 5 min after sample introduction and was then ramped (12 °C /min) to 240°C, with a 1 min hold, before ramping (30 °C/min) to 325 °C, which was held for 4 min prior to returning to the starting conditions.

The MS data files were pre-processed and analyzed with Genedata Expressionist software tools. Workflow included: nominal mass integration (centroids integrated to nominal mass value peaks), chromatogram blank subtraction, chemical noise subtraction, extracted ion

chromatogram alignment, retention indexing (ethyl ester; C2-C14), chromatogram nominal mass peak detection and component detection (library matching; NIST v 2.2). Statistical workup was performed in Genedata Analyst on the weight normalized intensity of the most abundant ion representing the deconvoluted EI fragment groups. K groups and analysis of variance (ANOVA) were performed between all three lines and 2 groups (group medians) were performed for comparison between the I3 line and the I5 or I7 lines portrayed as volcano plots. Principal component analysis (PCA) modeling was performed by PLS_Toolbox (Eigenvector Research, Inc.) within MATLAB and featured autoscaling to accommodate the magnitude in the MS signal dynamic range.

References

Bürkner, P. (2017). brms: An R Package for Bayesian Multilevel Models using stan. *J. Statist. Soft.* 80, 1-28.

Sinha, K., Kaur, R., Singh, N., Kaur, S., Rishi, V., Bhunia, R.K. (2020). Mobilization of storage lipid reserve and expression analysis of lipase and lipoxygenase genes in rice (Oryza sativa var. Pusa Basmati 1) bran during germination. Phytochemistry 180. https://doi.org/10.1016/j.phytochem.2020.112538

Vehtari, A., Gelman, A., and Gabry, J. (2017). Practical Bayesian model evaluation using leave-one-out cross-validation and WAIC*. Stat. Comput.,* 27, 1413–1432.

Supplementary Figures

Figure S1. TAG and FFA levels in milled flour under accelerated-ageing conditions compared across three experiments. TAG and FFA levels measured by HPLC-ELSD in two datasets (2019 and 2021; same data as Figure 2c) compared to a third dataset (NABI-2021) measured by TLC/GC-MS. Log-linear hierarchical mixed models were utilized to assess the differences in TAG and FFA levels of these three inbred lines. Points represent observed data, lines represent posterior mean of the predictive distribution, and the ribbon corresponds to the 95% credible interval of the posterior mean predictive distribution.

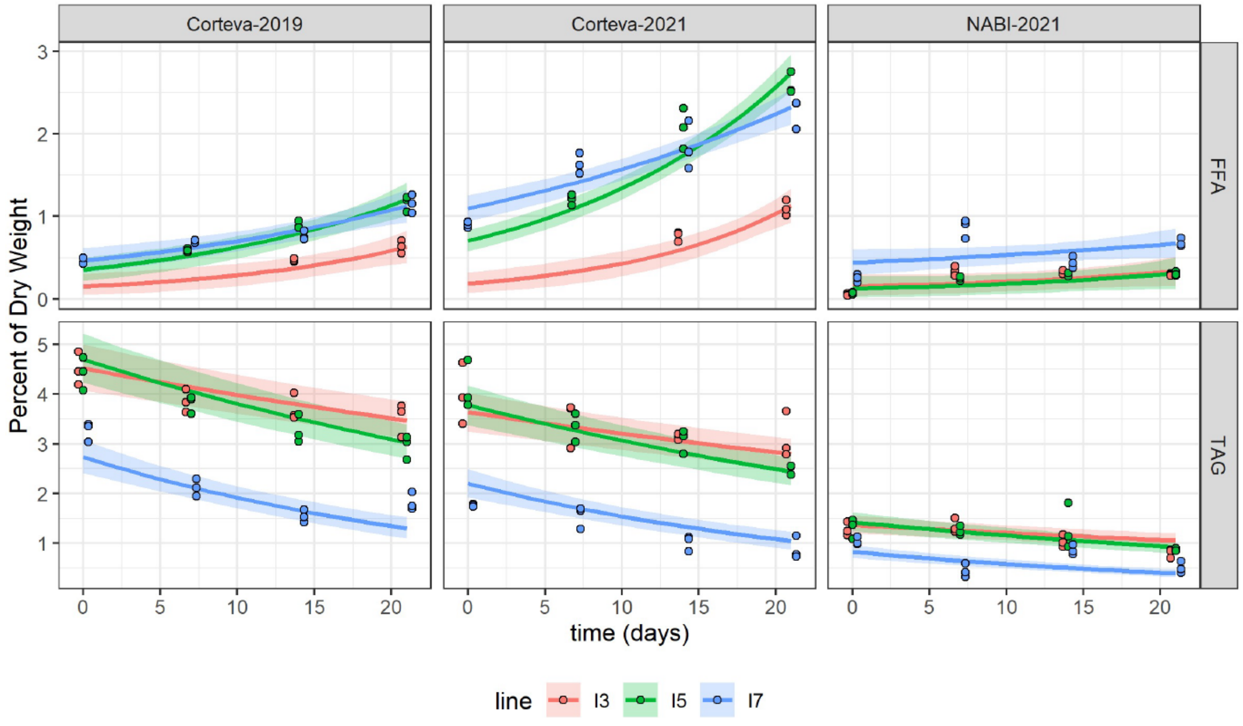

Figure S2. Sequence variations of selected TAG lipases. Comparative amino acid sequence alignment of three PgTAGLips, PgTAGLip1, PgTAGLip2 and PgTAGLip3 using the Clustal W program. The amino acids Ser (S), Asp (D) and His (H) residues forming the catalytic triad of TAG Lips are indicated with # symbol. The LID domain in bold letters carries the underlined lipase serine active motif (GxSxG). The putative N-glycosylation sites are indicated in red.

```
PgTAG Lip1  MDRRRRAVKAATVMVLL----LLSIDPAGGARRDWGFHSPPSVGFTQSNTEPDVRSSGKSFASNYTLARAIVEYASAVYM
PgTAG Lip2  -----MAAKGATAALLLLAALLVSAPPTA-------------AAAAEQKGVLTMKPSDGGYAYNRTLAHILVEYASAVYT
PgTAG Lip3  MGRWRCA---GVVALVL----LLSA--------------------ASHGRELPVKKSDHSFVYNHTLAKTIVEYASAVYM
                  *   ... ::*    *:*                     :.      :: *. .:. * ***: :********

PgTAG Lip1  TDLTALYTWTCSRCNDLTRGFEVTCIIVDVQNCLQAFVGVDHNPNAIIVAIRGTQENSIQNWIKDLIWKQVNLNYPNMPN
PgTAG Lip2  SDLTSLFTWTCPRCKDHTKGFEVIEVIVDVANCLQAFVGVAPDPQSIIIAFRGTQQHSVSNWIEDLFWKQLDVTYPGMPD
PgTAG Lip3  SDLTALYTWTCSRCNDLTQGFEMRSLIVDVENCLQAFVGVAHNLNSIVVAIRGTQENSVQNWIEDLVWKQLDLSYPNMPN
            :***:*:**** **:* *:***:  :**** *********: ::*::*:****::*:.***:**.***:::.**.**:*
                                                    #
PgTAG Lip1  AKVHIGFYSSYNNTLLRPAITNAVRKARKLHGDCDIIVTGHSMGGAIASFCALDLAISFGSDNVHLMTFGQPRVGNAAFA
PgTAG Lip2  AMVHHGFYTAYYNTTLRFEILKSIKWARKTYGKLPINVVGHSMGGALASFCALDLSVKFGSQEVELMTFGQPRIGNPAFA
PgTAG Lip3  AKVHRGFFSSYNNTILRLAITSAVRKARKSYGDINVIVTGHSMGGAMASFCALDLAMKLGSDRVQLMTFGQPRVGNAAFA
            ** **:::* ** **  *   .::: *** :*.  : *.*******:********::.:**:.*.********:** ***
                         #
PgTAG L1p1  SYFAKYVPNTIRMTHQRDIVPHLPPYFFFLPQLTYRHFPREVWEHDVDGK----TTFQVCDGSGEDPNCSRSVFVLFWSA
PgTAG Lip2  AYFAAQVPRTIRVTHQNDIVPHLPPYYYYLGEWTYHHFAREVWLHVIIDGNVITRNETICDDSGEDPTCSRSVY--GISV
PgTAG Lip3  SCFAKYVPNTIRVTHGHDIVPHLPPYFSFIPQWTYHHFPREVWVQDSNGN----TTVKICDDSGEDPHCCRSISMFSLSI
            : **  **.***:** .*********: :: : **:** **** :   .      .  :**.***** *.**:     *
                      #
PgTAG Lip1  TDHLTYMGVKIQADDWSTCRIVMGRSVE--QLQRSLASSITTSGLSIDVIIADNSVQVV*
PgTAG L1p2  ADHLEYYGVTMHSDSRGSCQFVMGSANSVYSYIREVDGTIILSKYAQESHILESI*
PgTAG Lip3  QDHFTYLGVDMQADDWSTCRIIAAESVK--QFRKDLTSNIIMAKHNADIFIVEPSVQTDRSSSR*
             **: * ** :::*. .:*::: . : .  .  :.: ..*  :    :  * :
```

Figure S3. (a) Quantification of intracellular yeast neutral lipid bodies using flow cytometry. The X-axis represent fluorescein isothiocyanate-A (FITC-A) mean fluorescence intensity, where Y-axis represents cell count showing fluorescence. Increased fluorescence indicates greater numbers of lipid bodies. (b) Expression of indicated TAG lipase variants in ΔTGL yeast strain alters TAG content accumulation identified by FACS. Each data point represents mean of two biological replications with standard error.

Figure S3

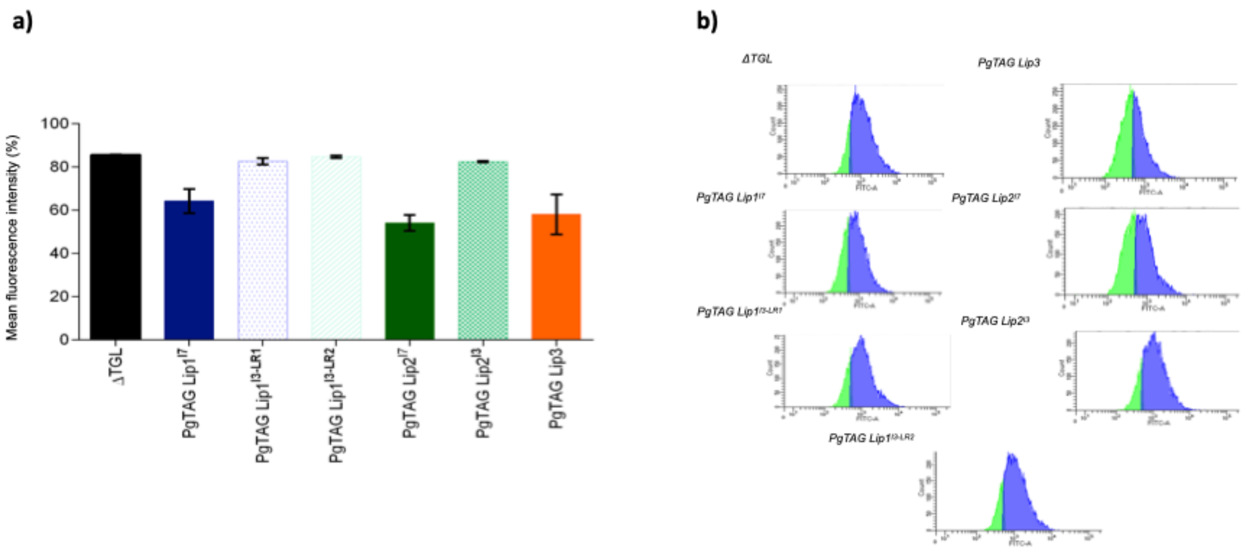

Appendix B. Supplementary Figures

Figure S1. TAG and FFA levels in milled flour under accelerated-ageing conditions compared across three experiments. TAG and FFA levels measured by HPLC-ELSD in two datasets (2019 and 2021; same data as Figure 2c) compared to a third dataset (NABI-2021) measured by TLC/GC-MS. Log-linear hierarchical mixed models were utilized to assess the differences in TAG and FFA levels of these three inbred lines. Points represent observed data, lines represent posterior mean of the predictive distribution, and the ribbon corresponds to the 95% credible interval of the posterior mean predictive distribution.

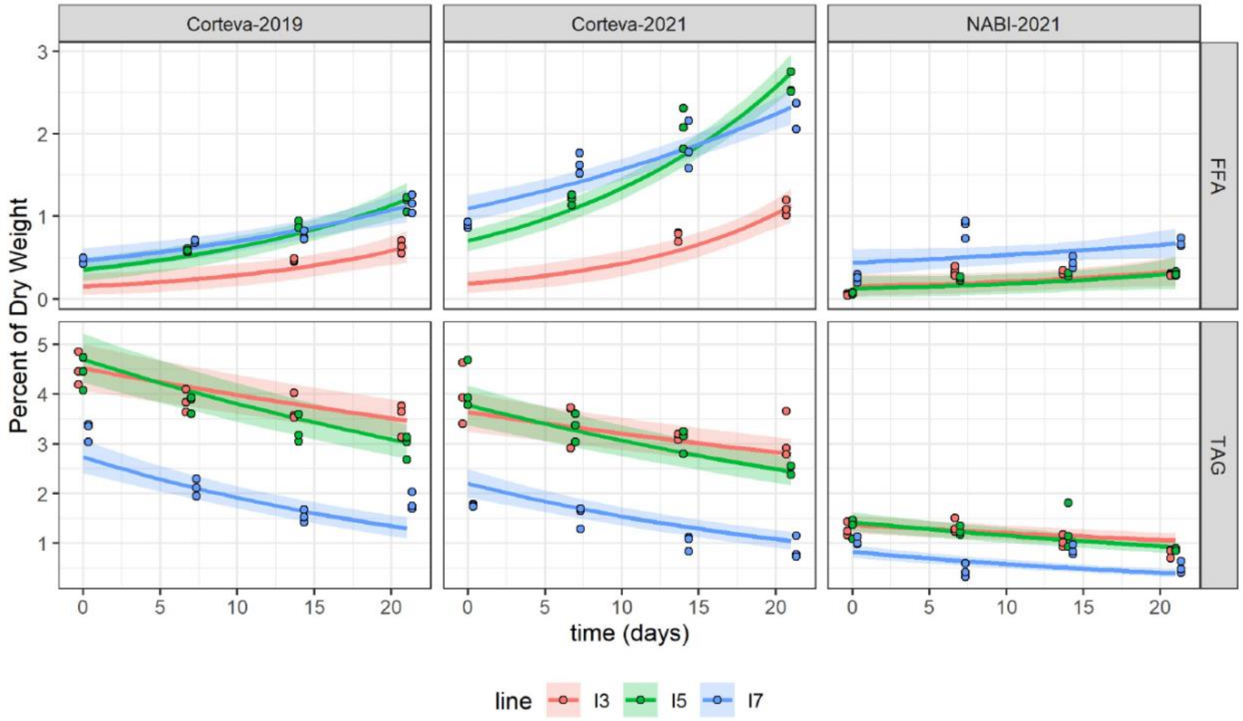

Figure S2. Sequence variations of selected TAG lipases. Comparative amino acid sequence alignment of three PgTAGLips, PgTAGLip1, PgTAGLip2 and PgTAGLip3 using the Clustal W program. The amino acids Ser (S), Asp (D) and His (H) residues forming the catalytic triad of TAG Lips are indicated with # symbol. The LID domain in bold letters carries the underlined lipase serine active motif (GxSxG). The putative N-glycosylation sites are indicated in red.

```
PgTAG Lip1   MDRRRRAVKAATVMVLL----LLSIDPAGGARRDWGFHSPPSVGFTQSNTEPDVRSSGKSFASNYTLARAIVEYASAVYM
PgTAG Lip2   -----MAAKGATAALLLLAALLVSAPPTA-------------AAAAEQKGVLTMKPSDGGYAYNRTLAHILVEYASAVYT
PgTAG Lip3   MGRWRCA---GVVALVL----LLSA--------------------ASHGRELPVKKSDHSFVYNHTLAKTIVEYASAVYM
                   *   ... ::*    *:*                     :.      :: *. .:. * ***: :********

PgTAG Lip1   TDLTALYTWTCSRCNDLTRGFEVTCIIVDVQNCLQAFVGVDHNPNAIIVAIRGTQENSIQNWIKDLIWKQVNLNYPNMPN
PgTAG Lip2   SDLTSLFTWTCPRCKDHTKGFEVIEVIVDVANCLQAFVGVAPDPQSIIIAFRGTQQHSVSNWIEDLFWKQLDVTYPGMPD
PgTAG Lip3   SDLTALYTWTCSRCNDLTQGFEMRSLIVDVENCLQAFVGVAHNLNSIVVAIRGTQENSVQNWIEDLVWKQLDLSYPNMPN
             :***:*:**** **:* *:***:  :**** *********: ::*::*:****::*:.***:**.***:::.**.**:*
                                                      #
PgTAG Lip1   AKVHIGFYSSYNNTLLRPAITNAVRKARKLHGDCDIIVTGHSMGGAIASFCALDLAISFGSDNVHLMTFGQPRVGNAAFA
PgTAG Lip2   AMVHHGFYTAYYNTTLRFEILKSIKWARKTYGKLPINVVGHSMGGALASFCALDLSVKFGSQEVELMTFGQPRIGNPAFA
PgTAG Lip3   AKVHRGFFSSYNNTILRLAITSAVRKARKSYGDINVIVTGHSMGGAMASFCALDLAMKLGSDRVQLMTFGQPRVGNAAFA
             ** **:::* ** **  *   .::: *** :*.  : *.*******:********::.:**:.*.********:** ***
                              #
PgTAG L1p1   SYFAKYVPNTIRMTHQRDIVPHLPPYFFFLPQLTYRHFPREVWEHDVDGK----TTFQVCDGSGEDPNCSRSVFVLFWSA
PgTAG Lip2   AYFAAQVPRTIRVTHQNDIVPHLPPYYYYLGEWTYHHFAREVWLHVIIDGNVITRNETICDDSGEDPTCSRSVY--GISV
PgTAG Lip3   SCFAKYVPNTIRVTHGHDIVPHLPPYFSFIPQWTYHHFPREVWVQDSNGN----TTVKICDDSGEDPHCCRSISMFSLSI
             : **  **.***:** .*********: :: : **:** **** :    .       .  :**.***** *.**:      *
               #
PgTAG Lip1   TDHLTYMGVKIQADDWSTCRIVMGRSVE--QLQRSLASSITTSGLSIDVIIADNSVQVV*
PgTAG L1p2   ADHLEYYGVTMHSDSRGSCQFVMGSANSVYSYIREVDGTIILSKYAQESHILESI*
PgTAG Lip3   QDHFTYLGVDMQADDWSTCRIIAAESVK--QFRKDLTSNIIMAKHNADIFIVEPSVQTDRSSSR*
              **: * ** :::*. .:*::: . : .  .  :.: ..*  :    :  * :
```

Figure S3. (a) Quantification of intracellular yeast neutral lipid bodies using flow cytometry. The X-axis represent fluorescein isothiocyanate-A (FITC-A) mean fluorescence intensity, where Y-axis represents cell count showing fluorescence. Increased fluorescence indicates greater numbers of lipid bodies. (b) Expression of indicated TAG lipase variants in ΔTGL yeast strain alters TAG content accumulation identified by FACS. Each data point represents mean of two biological replications with standard error.

Figure S3

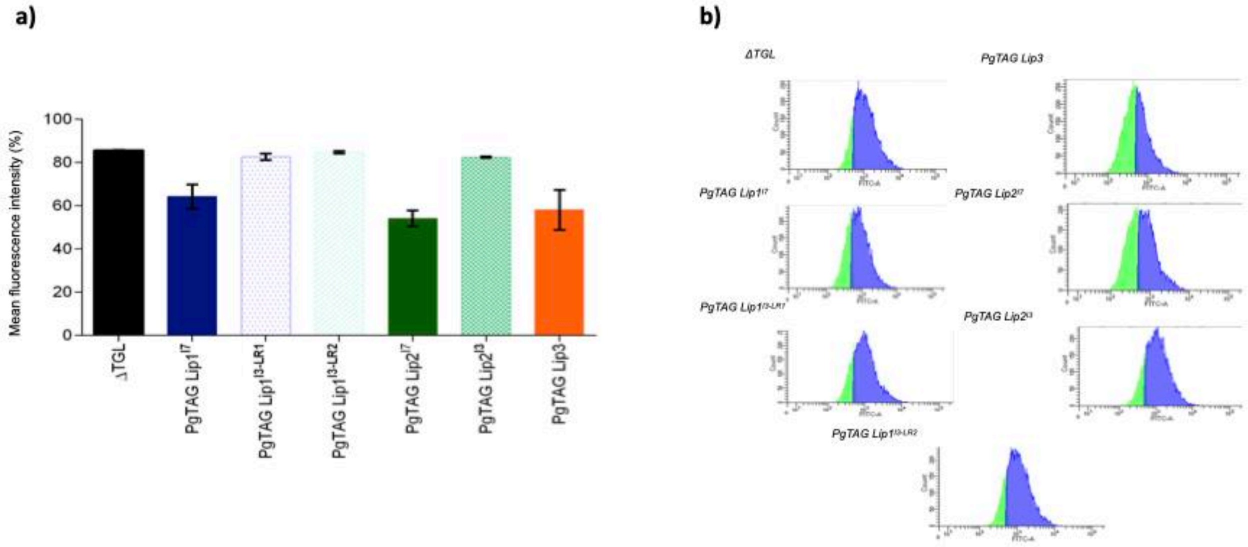

Appendix C. Supplementary Tables

Table S1: Identities and codes of pearl millet genotypes used in this study.

Genotypes	Codes
ICMB-843	I1
ICMB-88004	I2
ICMB-95222	I3
ICMB-81	I4
ICMB-89111	I5
ICMB-842	I6
ICMB- 863	I7
ICMB-98222	I8
IP5931	P13
IP 13840	P14
IP 22419	P19
IP 6099	P20

Additional genotypes used in molecular analysis based on Goswami et al., 2020.

Genotypes	Rancidity Potential
86M88	High
Super Boss	Low
RHB 177	Low

Table S2. List of the primers used for full-length gene cloning, qRT-PCR and yeast expression analysis.

S.No	Gene	Primers	Remarks
1	PgTAG Lip1 _F	ATGGATAGGCGGAGACGCGCGGT	Full-length gene cloning
2	PgTAG Lip1_R	TCAAACGACCTGGACGCTATTGTC	
3	PgTAG Lip2_F	ATGGCTGCGAAGGGCGCGAC	
4	PgTAG Lip2_R	TCATATGGATTCTAGGATATG	
5	PgTAG Lip3_F	ATGGGGAGATGGAGGTGCGC	
6	PgTAG Lip3_R	CTATCTAGAACTGCTCCGAT	
7	PgTAG Lip2_qF	GGGCTTTGAGGTGATTGAGGT	Expression analysis
8	PgTAG Lip2_qR	ACACTGTGCTGTTGAGTCCC	
9	PgTAG Lip3_qF	GCTGCAGGTCCATCTCCATG	
10	PgTAG Lip3_qR	TGCTGGTGAGATCCTTTCGG	
11	PgTAG Lip1 _qF	GCGTGTTTGTGCTGTTCTGG	
12	PgTAG Lip1_qR	TTGCCCCATGACGATTCTGC	
13	PgEIF4A_qF	ATCGTGAGCTTTACATCCATCG	
14	PgEIF4A_qR	TATCCCTCAGGATACGGATGTC	
15	PgMDH_qF	AGAAGGCGCTTGCTTACTCAT	
16	PgMDH_qF	CAGTTCTGGGTGAGGGAATCT	
17	PgTAGLip1_I7YF	ATTAGGTACCATGGATAGGCGGAGACGCGC	Yeast expression
18	PgTAGLip1_I7YR	CGCAGAGCTCTCAAACGACCTGGACGCTAT	
19	PgTAGLip1_I5YF	ATTAGGTACCATGGATAGGCGGAGACGCGC	
20	PgTAGLip1_I5YR	CGCAGAGCTCTCAGCTGTGGGAGGAAGAA	
21	PgTAGLip1_I3LR1_YF	ATTAGGTACCATGGATAGGCGGAGACGCGC	
22	PgTAGLip1_I3LR1_YR	CGCAGAGCTCTTATGTCACAATCTCCATG	
23	PgTAGLip1_I3LR2_YF	ATTAGGTACCATGGATAGGCGGAGACGCGC	
24	PgTAGLip1_I3LR2_YR	CGCAGAGCTCTCAAACCCATCTTGAGCAT	
25	PgTAG Lip2_I7_YF	ATTAGGTACCATGGCTGCGAAGGGCGCG	
26	PgTAG Lip2_I7_YR	CGCAGAGCTCTCATATGGATTCTAGGAT	
27	PgTAG Lip2_I5_YF	ATATAAGCTTATGGATAGGCGGAGACGCGC	
28	PgTAG Lip2_I5_YR	AGATGGATCCTCCTAAACCTCTCTAGCAAA	
29	PgTAG Lip3_YF	ATTAGGTACCATGGGGAGATGGAGGTGC	
30	PgTAG Lip3_YR	CGCAGAGCTCCTATCTAGAACTGCTCC	

---Internal Use---

Table S3. Sensory evaluations of the milled flour from lines i3, i5, and i7 during 21 days of storage under accelerated aging conditions.

Days of storage	Odour				Taste		
	I3	I5	I7		I3	I5	I7
0	Dry flour	Cooked rice	Pungent		Spoiled rice	Bitter gourd	Bitter
	Dry flour	Fresh dry flour	Light acid smell		Bitter	Light bitter	Slightly bitter
	Wheat dough	Dry flour	Slightly flour like smell		Slightly sweet	Flour like	Bitter
1	Sweet	Dry flour	Light pungent		Bitter	Bitter	Bitter
	Mild flour	Wet mud	Stale dough		Sweet wheat flour		Slightly bitter
	Fermented dough	Sweet	Pungent		Tasteless	Slightly bitter	Bitter
7	Fermented batter	Light gym socks	Stored rice flour		Sour	Bitter	Camphor
	Sweet	Fermented dough	Foul		Bitter	Bitter	Bitter
	No odour	Pungent	Damp-dough		Slight bitter	Bitter corn	Bitter
14	Strong wheat flour	Stored rice	Gym socks		Bitter as bitter gourd	Slightly bitter	Chalk powder
	No pungency	Slightly stale wheat	Strong bad smell		No taste	Bitter	Mud like
	Sweet smell	Bad smell	Woody, wet soil, tree bark smell		Sour	Bitter	Bitter
21	General flour smell	Foul	Stored rice bags		Bitter and sour	Stored cornflakes	Stored wheat flour
	Very light pungent	Very light pungent	Light pungent		No Taste	Very bitter	Spoiled flour taste
	Strong flour	Pungent	Damp/woody		Light bitter	Bitter	Mud like

Table S4. Pairwise contrasts of line TAG (left) or FFA (right) values shown in Figure 2c. Pairwise median posterior estimates of differences between (log) TAG values for each line pair at each time point along with 95% highest probability density credible interval.

TAG						FFA					
**Days**	**contrast**	**Estimate**	**lower.HPD**	**upper.HPD**	**sig**	**Days**	**contrast**	**Estimate**	**lower.HPD**	**upper.HPD**	**sig**
**0**	I3 - I5	-0.026	-0.118	0.069	-	**0**	I3 - I5	-1.302	-2.05	-0.685	*
	I3 - I7	0.564	0.44	0.692	*		I3 - I7	-1.722	-2.43	-1.088	*
	I5 - I7	0.59	0.469	0.718	*		I5 - I7	-0.421	-0.646	-0.193	*
**7**	I3 - I5	0.04	-0.042	0.115	-	**7**	I3 - I5	-1.146	-1.619	-0.724	*
	I3 - I7	0.732	0.631	0.833	*		I3 - I7	-1.377	-1.857	-0.974	*
	I5 - I7	0.693	0.59	0.791	*		I5 - I7	-0.232	-0.385	-0.083	*
**14**	I3 - I5	0.106	0.022	0.191	*	**14**	I3 - I5	-0.991	-1.252	-0.771	*
	I3 - I7	0.9	0.767	1.028	*		I3 - I7	-1.033	-1.296	-0.813	*
	I5 - I7	0.795	0.667	0.926	*		I5 - I7	-0.042	-0.144	0.058	-
**21**	I3 - I5	0.171	0.058	0.276	*	**21**	I3 - I5	-0.832	-1.022	-0.65	*
	I3 - I7	1.067	0.887	1.269	*		I3 - I7	-0.682	-0.877	-0.494	*
	I5 - I7	0.896	0.71	1.094	*		I5 - I7	0.148	0.033	0.263	*

---Internal Use---

Table S5. Statistical analysis for the SPME-EI-GC data shown in Figure 3. Analysis of variance (ANOVA) from K Groups (group medians) sample comparison tests for the four most abundant aldehydes in the mass spectral variable set. *p*-values and adjusted *p*-values (Benjamini Hochburg (BH Q-Value) are shown.

**Analysis of Variance**	***p*-Value**	**BH Q-Value**
hexanal	6.00E-07	2.00E-03
benzeneacetaldehyde	2.20E-06	3.60E-03
octanal	1.30E-05	1.30E-02
nonanal	9.00E-05	3.00E-02
heptanal	2.60E-04	4.90E-02
pentanal	4.80E-04	6.70E-02
decanal	7.50E-01	9.40E-01

Table S6. List of the subfamily II lipase genes identified in this study and genomic and protein characteristics. Common properties such as open reading frame (ORF), amino acid translations, molecular weight (kDa) and isoelectric point (pI) of each pearl millet TAG Lipase (PgTAGLip) were calculated using the MacVector software (V17.1). Genes in bold were named and further characterised.

Gene Accession No.	Putative functional annotation	Lip domain/GXSXG motif	Major regions/domain	Nucleotide			Protein			
				ORF size	Chromosome	Chromosome location	Amino acids	MW	pI	Subcellular localization*
Pgl_GLEAN_10007548	phospholipase A1-II 3	ITITGHSLGG (106 - 115)/GXSXG	lipase class 3	792	chr6	104623040:104626472	263	28	5.04	chloroplast
Pgl_GLEAN_10007551	phospholipase A1-II 2-like	ITVVGHSLGA (160 - 169)/GXSXG	lipase class 3	1059	chr6	104679622:104682716	352	39.2	5.7	cytosol
Pgl_GLEAN_10007552	phospholipase A1-II 1	ITITGHSLGA (219 - 228)/GXSXG	PPR_2/lipase class 3	1185	chr6	104685154:104687393	394	44.07	6.33	mitochondria
Pgl_GLEAN_10021604	phospholipase A1-II 7-like	ITITGHSLGA (11 - 20)/GXSXG	lipase class 3	582	chr1	258491452:258492033	193	20.84	5.62	endoplasmic reticulum
Pgl_GLEAN_10008833	Phospholipase A1-IIgamma	ITVTGHSLGA (122 - 131)/GXSXG	lipase class 3	588	chr1	233144775:233146216	195	21.56	5	chloroplast
Pgl_GLEAN_10000987	uncharacterized protein		Lipase_3/catalytic triad	672	chr6	chr6:107492484:107494611	223	24.46	9.54	nucleus
Pgl_GLEAN_10008103	phospholipase A1-Ibeta2	ITVVGHSLGA (142 - 151)	triacylglycerol lipase/PLN02802/catalytic triad	981	chr3	34308672:34309652	326	35.33	8.68	chloroplast
Pgl_GLEAN_10021768	phospholipase A1-Ibeta2/	ITIVGHSLGA (153 - 162)	triacylglycerol lipase/PLN02802/catalytic triad	-	chr5	99694060:99695920	-	-	-	chloroplast
Pgl_GLEAN_10005797	phosphoinositide phospholipase C 2-like		PA2_HIS	354	chr2	chr2:18365615:18367043	117	12.72	5.48	extracellular
Pgl_GLEAN_10011237	lipase like		Lipase_3/catalytic triad	1029	chr1	163462080:163464746	342	38.24	5.84	extracellular
Pgl_GLEAN_10024115	lipase like	INVVGHSMGG(178 - 187)	Lipase_3/catalytic triad	1068	chr1	7199095:7202565	355	39.29	5.94	extracellular
Pgl_GLEAN_10023115	lipase-like	IIVTGHSMGG (192-201)	Lipase_3/catalytic triad	1110	-	-	370	34.88	8.77	vacuole
Pgl_GLEAN_10011213	lipase like	VIVTGHSMGG (169 - 178)	Lipase_3/catalytic triad	1056	chr7	1341282:1344315	351	39.29	7.63	chloroplast
Pgl_GLEAN_10036216	monoglyceride lipase	GXSXG (118-122)	hydrolase; alpha/beta fold family protein	909	chr6	234243736:234247360	302	33.33	6.9	cytosol

Table S7. Proteins encoded by selected subfamily II lipases in pearl millet, their docking score, and observed hydrogen bonds between various docked substrates (triglycerides). The lower the score, the greater the preference for that substrate.

Gene Name	pNPA	Triacetin	Triarachidin	Tricaprin	Trilinolein	Triolein	Tripalmitin	Tristrearin
Pgl_GLEAN_10023115 (*PgTAGLip1*)	-3.45	-2.72	-11	0	-1.9	-4.5	-2.5	-2
Pgl_GLEAN_10024115(*PgTAGLip2*)	-3.14	-2.2	-10.79	-1.2	-8.2	-4.1	-0.6	-0.9
Pgl_GLEAN_10011213(*PgTAGLip3*)	-2.9	-2.3	-11.4	-2.5	-8.7	-3.5	-0.5	-0.9
Pgl_GLEAN_10008103	-3.45	-3.5	-10.8	-5	-7.2	-3.8	-0.5	-0.28
Pgl_GLEAN_10007551	-6.64	-4.84	-7.64	-2.72	-3.42	-2.7	-4.44	-3.83
Pgl_GLEAN_10021604	-5.95	-4.35	-9.39	-1.35	-3.23	-1.1	-3.93	-5.32
Pgl_GLEAN_10007548	-6.85	-5.15	-5.17	-0.3	-3.69	-0.86	-4.85	-5.99
Pgl_GLEAN_10007552	-6.11	-3.6	-7.97	-0.61	-3.04	-0.71	-4.19	-3.6
Pgl_GLEAN_10008833	-6.27	-4.67	-7.82	-1.09	-2.11	-0.66	-3.07	-3.46
Pgl_GLEAN_10011237	-5.9	-4.28	8.73	-1.04	0.2	-0.82	1.26	5.84
Pgl_GLEAN_10036216	-6.54	-4.84	7.24	-2.72	3.22	-2.7	4.44	3.83

---Internal Use---

Table S8. Allelic variations found in of high (I7, 86M88, I5) and low rancid lines (I3 (LR1, LR2, Super Boss, RHB177) of Pg*TAGLip1* and Pg*TAGLip2.*

Gene	Genotype	ORF size (Nucleotides)	Protein size (Amino acids)	Lid domain/GXSSG motif/Catalytic triad
PgTAG Lip1	I7	1110	370	Present
	86M88	1110	370	Present
	I5	807	269	Histidine residue absent
	I3 LR1	528	176	Absent
	I3 LR1	276	92	Absent
	Super boss	420	140	Absent
	RHB177	285	95	Absent
	RHB223	276	92	Absent

Gene	Genotype	ORF	Protein	LID domain/GXSSG motif/Catalytic triad
PgTAG Lip2	I7	1068	356	Present
	I5	804	268	Present
	I3	1008	336	Present
